# Stem Cell Derived Organoids in Human Disease and Development

**DOI:** 10.1155/2019/7919427

**Published:** 2019-11-07

**Authors:** Stefan Liebau, Holger A. Russ, Alexander Kleger

**Affiliations:** ^1^Institute of Neuroanatomy & Developmental Biology, Eberhard Karls University Tübingen, Tübingen, Germany; ^2^Barbara Davis Center for Diabetes, University of Colorado-Anschutz Medical Campus, USA; ^3^Department of Internal Medicine I, University of Ulm, Albert-Einstein-Allee 11, 89081 Ulm, Germany

The advent of organotypic cell culture systems provides a roadmap to successfully set up personalized medicine approaches. Organotypic cultures, also referred as organoids, can be defined as three-dimensional (3D) structures derived either from pluripotent or organ-restricted stem cells harboring the ability to mimic *in vivo* architecture and multilineage differentiation of terminally differentiated tissues. Their ability for extended periods of self-renewal distinguishes them from previous “sphere” culture assays. The latter have already been reported over the last decades, particularly as tumor sphere cultures. Organoid models from different sources may fulfill a variety of the critical prerequirements needed in current molecular oncology, personalized medicine, and tissue engineering approaches. The unique capability to provide a 3D structure that effectively mimics the complexity of a given organ offers several advantages: (i) organoids are highly expandable, (ii) can form virtually every tissue, (iii) can recapitulate malignant transformation, and (iv) undergo successful transplantation and maturation *in vivo* as shown at least for some tissues such as the intestine. The basis for these advantages is provided by specifically tailored rather complex *in vitro* culture conditions, which aim to mirror the *in vivo* environment. This allows for even clonal expansion of adult stem cells to rebuilt diverse organ-specific cell types, effectively recapitulating human tissue architecture. On top, the associated stem cell hierarchy responsible for rapid cell turnover and tissue homeostasis remains preserved and allows functional studies of this hierarchy. The best studied organoid system originates from a single LGR5+ intestinal stem cell to self-expand as a cryptvillus-like structure. Dozens of studies meanwhile provided proof of concept that such intestinal organoids indeed fulfill all of the requirements mentioned herein. Meanwhile, similar studies were described for other organs, including the pancreas, the lung, and the brain. Thus, it is not surprising that “organoids” have ranked as the Method of the Year 2017. The pioneering researcher in the field has been Dr. Hans Clevers, whom with his colleagues, has foreseen the method's capacity and clinical value. Based on promising preclinical results, some of the tools have been already incorporated into a company called “HUB-Foundation Hubrecht Organoid Technology.” HUB offers access to organoids in a living biobank format for preclinical drug discovery and validation (http://hub4organoids.eu). Future studies have to prove the true value of these efforts to improve patient care in real-time. In sum, organoids are not only suitable for disease modelling and drug validation but also suitable for studying tissue development, regeneration, and niche organization across several organs and stem cell niches.

In this special issue, we welcomed review and original articles with a focus on organoids derived either from adult stem cell compartments or differentiated from pluripotent stem cells. Additionally, we encouraged reports on “disease-specific organoid papers” and their specific advantages to model diseases, test drugs, and assess developmental changes. Four complementary review articles were accepted for publication in this special issue, focusing on the advances and opportunities of organoid technology within different endodermal tissues. J. Kraiczy and M. Zilbauer outline the use of epithelial organoids to study the underlying epigenetics of healthy and disease intestinal epithelial organoids, while L. Schulte and coworkers focus on the use of intestinal organoids to dissect inflammatory bowel disease. U. Tharehalli and colleagues outline the improvements in modeling liver carcinogenesis using organoids, and M. Hohwieler et al. exemplify how to model pancreatic diseases and cancer using this technology. In addition to the review articles, seven original research articles have been selected for publication. The research work presented spans the range of organoid research activities summarized in the review articles. Four papers focus on pancreatic diseases: A. Hennig and colleagues present evidence for the use of CTFR expression to classify human pancreatic cancer organoids, while S. Alcalá et al. demonstrate that the anthrax toxin receptor 1 is enriched in stem cell-enriched subpopulation of this cancer. In another study, the Herman laboratory shows that inhibition of the mitogen-activated protein kinase and extracellular signaling-regulated kinase (MEK) pathway mitigates migration of pancreatic cancer cells, indicating a potential treatment modality. Lastly, L. Perkhofer and associates show the Kras dependence of pancreatic ductal organoids for tumorigenic transformations. J. H. Jee and affiliates described a novel collagen-based matrix culture system for gastrointestinal tract-derived organoids. Z. Zhu et al. use intestinal organoids to provide compelling evidence that bacitracin can protect intestinal epithelial cells from the Clostridium difficile toxin TcdB. The study of L. Nguyen et al. is of particular interest, in which the authors describe the use of an isogenic three-dimensional nephron progenitor model that incorporates mesenchymal, endothelial, and renal progenitor cells. Taken together, this special issue provides a comprehensive collection of original research work utilizing these technologies for research advancements and is complemented by timely review articles on the subject summarizing recent advances of organoid cultures.

